# Whole Genome Sequencing of Chinese White Dolphin (*Sousa chinensis*) for High-Throughput Screening of Antihypertensive Peptides

**DOI:** 10.3390/md17090504

**Published:** 2019-08-28

**Authors:** Kuntong Jia, Chao Bian, Yunhai Yi, Yanping Li, Peng Jia, Duan Gui, Xiyang Zhang, Wenzhi Lin, Xian Sun, Yunyun Lv, Jia Li, Xinxin You, Qiong Shi, Meisheng Yi, Yuping Wu

**Affiliations:** 1 Southern Marine Science and Engineering Guangdong Laboratory (Zhuhai),Guangdong Provincial Key Laboratory of Marine Resources and Coastal Engineering, Zhuhai Key Laboratory of Marine Bioresources and Environment, School of Marine Sciences, Sun Yat-Sen University, Zhuhai 519082, China; 2 Shenzhen Key Lab of Marine Genomics, Guangdong Provincial Key Lab of Molecular Breeding in Marine Economic Animals, BGI Academy of Marine Sciences, BGI Marine, BGI, Shenzhen 518083, China

**Keywords:** Chinese white dolphin (*Sousa chinensis*), whole genome sequencing, genome assembly, antihypertensive peptide

## Abstract

Chinese white dolphin (*Sousa chinensis*), also known as the Indo-Pacific humpback dolphin, has been classified as “Vulnerable” on the IUCN Red List of Threatened Species. It is a special cetacean species that lives in tropical and subtropical nearshore waters, with significant differences from other cetaceans. Here, we sequenced and assembled a draft genome of the Chinese white dolphin with a total length of 2.3 Gb and annotation of 18,387 protein-coding genes. Genes from certain expanded families are potentially involved in DNA replication and repairing, suggesting that they may be related to adaptation of this marine mammal to nearshore environments. We also discovered that its historical population had undergone a remarkable bottleneck incident before the Mindel glaciation. In addition, a comparative genomic survey on antihypertensive peptides (AHTPs) among five representative mammals with various residential habitats (such as remarkable differences in exogenous ion concentrations and sea depth) revealed that these small bioactive peptides were highly conserved among these examined mammals, and they had the most abundant hits in collagen subunit proteins, especially for two putative AHTP peptides Gly-Leu-Pro (GLP) and Leu-Gly-Pro (LGP). Our genome assembly will be a valuable resource for further genetic researches on adaptive ecology and conservation biology of cetaceans, and for in-depth investigations into bioactive peptides in aquatic and terrestrial mammals for development of peptide-based drugs to treat various human cardiovascular diseases.

## 1. Introduction

Chinese white dolphin (*Sousa chinensis*), also known as the Indo-Pacific humpback dolphin, is a special cetacean species under the genus *Sousa* with a wide distribution from eastern India throughout Southeast Asia to central China. In many regions of China, it commonly inhabits estuaries and inshore waters, but it has more recently become an endangered species. It is believed that a total of six putative populations exist in China, including Xiamen (*n* = 86), the Pearl River Estuary (PRE) (*n* = 2,637), the Leizhou (*n* = 1,485), the Beibu Gulf (>89), the west coast of Taiwan (*n* = 99) and the west coast of Hainan [1,2,3]. Unfortunately, the total number of this marine mammal has declined gradually in the past decades. Our recent demographic studies in coastal waters of the Pearl River Delta region of China indicated a declining trend of ~2.5% per annum, suggesting that ~74% of its current population would probably disappear within the lifespan of three generations (~60 years) [4]. Thus, this cetacean species has been recently classified as “Vulnerable” on the IUCN Red List of Threatened Species [2,4].

Two major events, climate change and tectonic shifts, have made great contributions to the genetic diversity and structure of marine organisms [5,6]. It is known that numerous changes in the morphological, physiological and behavioral systems of cetaceans, such as *S. chinensis*, have been acquired to adapt to this drastic habitat transition from terrestrial to aquatic environments during the long-term evolution of cetaceans [7,8,9,10,11,12,13]. Thus, getting insight into the biological characteristics of cetaceans will benefit the understanding of the evolution of cetaceans and mechanisms for their adaptation to aquatic environments. The white dolphin provides a good example of speciation due to its special biological characteristics. For example, previous studies have shown that this species is primarily distributed in tropical and subtropical waters, and it resides in waters with depths of less than 20 m. It is also famous for the pink skin in adulthood. Similar to other cetaceans in adaptation to the marine environments, its body structure and physiology had exhibited dramatic changes, including streamlined bodies, absence of hind legs and outer ear pinnae, as well as presence of a fluke and blowhole [14,15]. However, the genetic backgrounds that underlies these biological properties are still limited.

Several previous studies showed that genomic analyses of marine mammals provided insights into molecular adaptation to living conditions [16,17]. For example, a comparative genomic analysis of walrus, bottlenose dolphin, killer whale, and manatee determined many convergent amino acid substitutions in these genomes and a portion of these substitutions were in several genes associated with a marine phenotype [16]. In the genome of the minke whale, a number of genes related to stress responses and anaerobic metabolism were expanded, while many genes related to body hair and sensory receptors were contracted [17].

However, the genetic mechanisms of *S. chinensis* underlying adaptation to the aquatic lifestyle is poorly understood. There are only few publications on its genetics and genomics. In the past decade, genetics studies have mainly focused on a single gene or a few genes, such as the major histocompatibility complex class II [18], CYP1A1 and HSP70 [19], as well as the mitochondrial DNA [20]. Recently, we provided the first transcriptome exploration of *S. chinensis* and identified a large number of genes related to adaptive evolution and cetacean-specific traits [9]; the first genome assembly of this marine mammal was also reported previously [21]. One more interesting question is how to regulate blood pressure for cetaceans in different sodium levels and sea depths. Angiotensin-converting enzyme 2 (ACE2), an important enzyme converting vasoconstrictor angiotensin II (Ang II) into inactive Ang (1–7), has been reported to experience changes in adaptation to freshwater [17]. Antihypertensive peptides (AHTPs) have a similar function as ACE2, while they mostly inhibit the production of angiotensin thus leading to the lowering of blood pressure [22]. Many AHTPs, mainly consisting of 2~10 amino acids, are usually digested from products of natural organisms [23]. Endogenous AHTPs may also result from hydrolyzation and degradation of in vivo proteins by certain enzymes, therefore acting as regulators of the renin angiotensin system by binding to angiotensin converting enzymes or related receptors [24]. However, the knowledge about changes of AHTPs in various mammals or cetaceans is still scarce, and there are few studies available for identification of potential protein types that can be hydrolyzed into large amount of AHTPs.

In this study, we report a de novo assembly and primary analyses of the Chinese white dolphin genome based on a cell-line sample that was previously derived from the skin of a dolphin captured by us [25]. Transcriptome sequencing was performed on extracted leucocytes from collected blood samples for assistance to genome annotation. Moreover, a comparative study on AHTPs was performed at a whole proteome level among five representative mammals with different living habitats, including the terrestrial cow, marine minke whale, epipelagic white dolphin and bottlenose dolphin, as well as the autopotamic Yangtze River dolphin. Here, we aim at providing a valuable genomic resource for genome-wide studies on cetaceans and an in-depth exploration of bioactive peptides for potential development of AHTP-based marine drugs.

## 2. Results

### 2.1. Summary of Genome Assembly and Annotation

**Figure 1:**
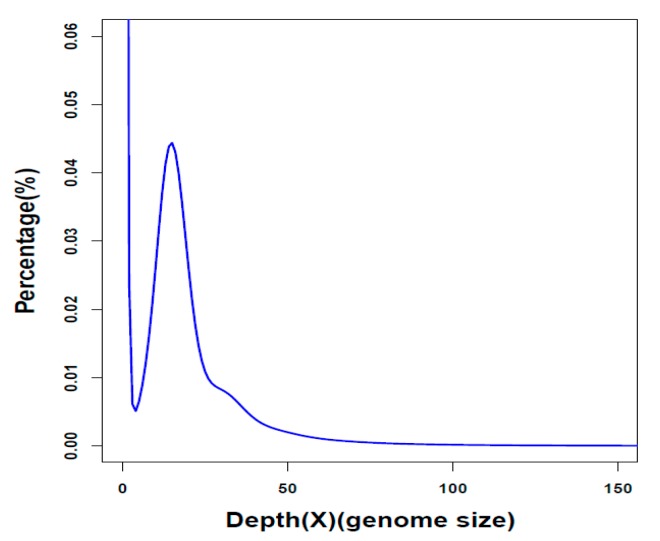
A *K*-mer analysis of the Chinese white dolphin genome. In our present study, the *k*-mer depth is 15, and the estimated genome size is ~2.6 Gb.

We generated a total of 318.4 gigabases (Gb) of raw reads (Table S1) by sequencing of seven libraries (see more details in Section 4.2.) in an Illumina HiSeq 2500 platform (Illumina, San Diego, CA, USA). After removal of low-quality reads, we obtained 245.9 Gb of clean data (Table S1), and assembled a 2.3 Gb genome of the Chinese white dolphin, which is close to the estimated genome size (~2.6 Gb) from a *k*-mer analysis (Figure 1). Our genome assembly is composed of 1789 scaffolds (>2000 bp), with a scaffold N50 of 19.2 Mb and a contig N50 of 84.3 Kb (Table 1). Although a primary genome assembly of *S. chinensis* was available, reported by Ming et al. [21], its scaffold N50 (163 kb) and contig N50 (12.9 kb) were shorter. Therefore, in this study, we improved the genome work with a high-quality assembly.

We further utilized the routine BUSCO (Benchmarking Universal Single-Copy Orthologs) method [26] to check the completeness of our genome assembly, determining that 95% were complete and partial eukaryote BUSCO orthologues. We also identified that repeat sequences account for about 42.3% of the assembled genome (Table S2), and annotated a final complete gene set of 18,387 genes with an average of 44.2 kb in length (Table 1). Approximately 93.9% of the predicted genes have at least one related function assignment from several public databases, including TrEMBL, SwissProt, KEGG and InterProScan (Table S3).

### 2.2. Genome Analyses

#### 2.2.1. Phylogenetic Analysis and Divergence Times

**Figure 2:**
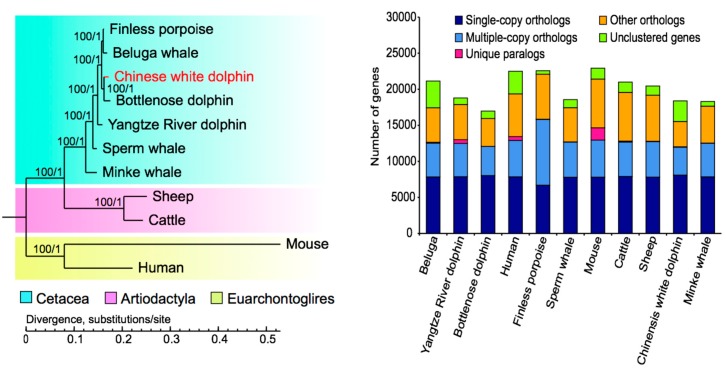
Phylogenetic relationships of Chinese white dolphin and other ten examined mammals (left) in component genes (right). This evolutionary topology indicates a clear division of three major groups of Cetacea, Artiodactyla and Euarchontoglires.

A phylogenetic tree was constructed to categorize eleven examined mammals (see more details in Section 4.4) into three major groups (left panel in Figure 2), which is consistent with the traditional taxonomic classification of Cetacea, Artiodactyla and Euarchontoglires. Among these groups, Cetacea appears to have a closer relationship with Artiodactyla than Euarchontoglires, which is strongly supported by the robust node confidence level. Our phylogenetic analysis of 5728 rigorously screened gene orthologs concurred with a recent online phylogenomic report [21] and previous research based on mitochondrial genomes [27].

Our divergence time analysis suggested that the cetaceans diverged from Artiodactyla about 53.4 million years ago (Mya; see Figure S1), which is consistent with previous reports [21,25]. Another previous study [28] also considered that the group of cetaceans had diverged from their terrestrial ancestors about 53~56 Mya when they reinvaded aquatic environments. We predicted that the Chinese white dolphin split with the bottlenose dolphin about 7.6 Mya (Figure S1), and since then it began to colonize estuaries and coastal areas. Our present phylogenomic data provide more evidences for the patterns of divergence and evolution in the group of cetaceans.

#### 2.2.2. Expansion and Contraction of Gene Families

**Figure 3:**
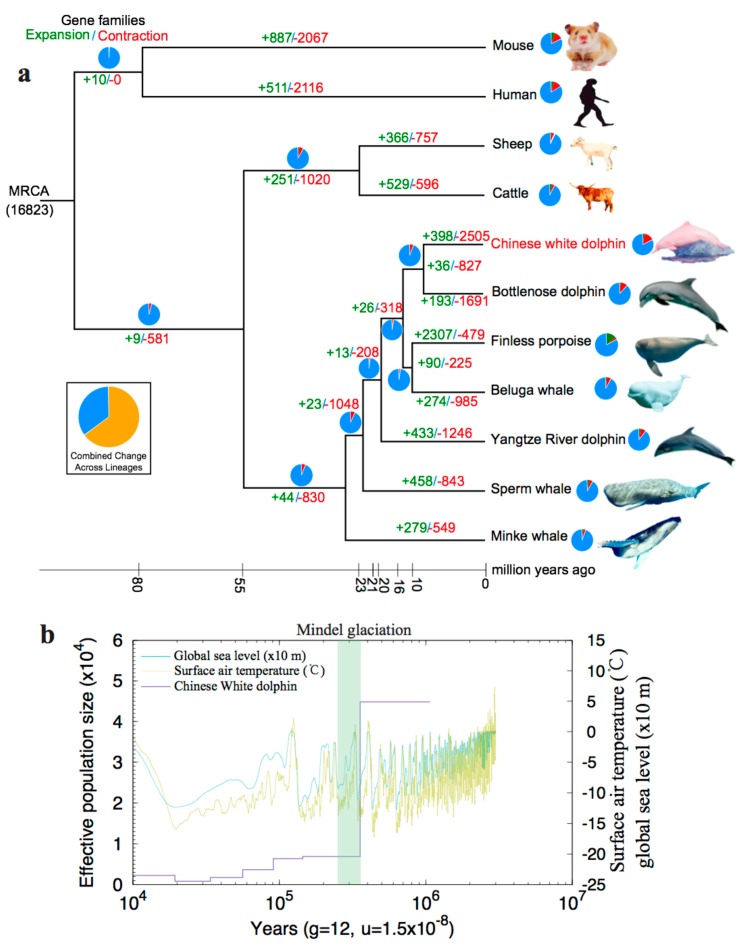
Evolution of the Chinese white dolphin. (**a**) A phylogenetic tree of the eleven examined mammals. Numbers associated with each branch stand for gene families that have expanded (green) or contracted (red) since splitting from the common ancestor. (**b**) Predicted population history of the Chinese white dolphin (see more explanations in Section 2.2.3). The purple line represents the population changes. The green and light-yellow lines denote the reported fluctuations of global sea level and surface air temperature. The green box represents the Mindel glaciation period.

**Figure 4:**
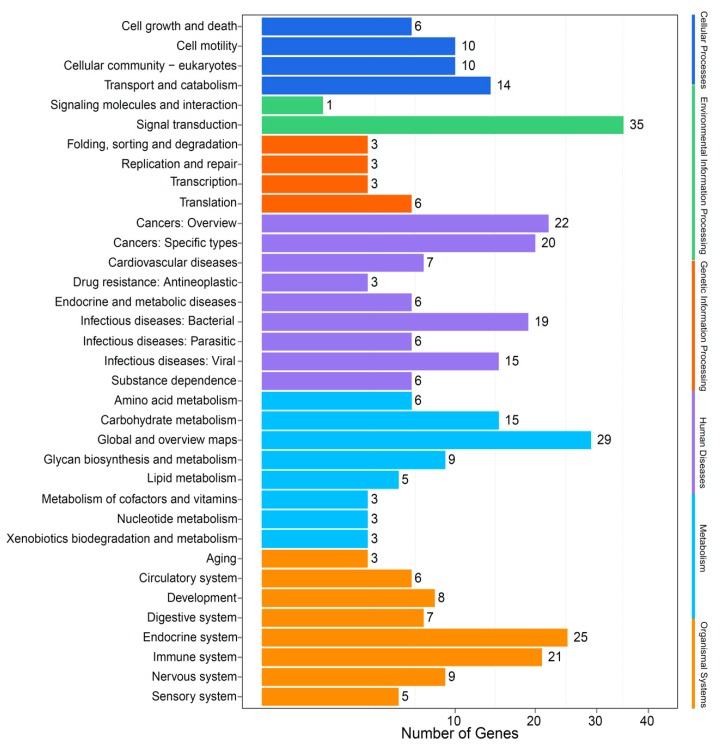
KEGG annotation of the expanded gene families in the Chinese white dolphin.

We determined the expansion and contraction of gene families (398 and 2505, respectively) in the Chinese white dolphin (see more details in Section 4.6). In comparison with other examined mammals, the largest number of the contracting gene ortholog cluster was inferred from the Chinese white dolphin (Figure 3a), suggesting a possible loss of many gene families during the evolution of this marine mammal. On the other hand, the 398 gene families expanded in the Chinese white dolphin are mainly involved in cell growth and death, transport and catabolism and lipid metabolism (see related annotation in Figure 4). Interestingly, certain genes involved in the nervous and sensory systems were expanded, which is consistent with the fact that the Chinese white dolphin is sensitive to anthropogenic pressures such as vessel traffic, coastal harbor construction, underwater blasting or dredging, extensive mariculture and fishing activities, as well as agricultural or industrial pollutants in the nearshore environments [21,29,30]. We also identified that certain gene families involved in DNA replication and repairing were also expanded, which may benefit this marine mammal to cope with a high UV exposure condition in the shallow waters.

#### 2.2.3. Population History

We utilized the PSMC approach [31] to reconstruct the population history of the Chinese white dolphin (see more details in Section 4.7). Interestingly, we observed a remarkable bottleneck incident in its historical population that appeared about 0.35 Mya (Figure 3b). Similarly, this phenomenon of a population bottleneck was previously reported in sperm whales and finless porpoises [32,33]. However, the corresponding period of population bottleneck for the Chinese white dolphin was much later (about one Mya) than those of the two reported marine mammals. 

After combining the reported data of atmospheric surface air temperature and global relative sea level (from the National Climatic Data Center at http://www.ncdc.noaa.gov/), we observed that the population of Chinese white dolphin had sharply declined after a remarkable reduction in temperature and sea level before the Mindel glaciation (middle in Figure 3b). Subsequently, the population size gradually decreased from 0.15 to 0.20 Mya, and this change pattern was similar to the curves of temperature and sea level during this critical period. Therefore, we reach a primary conclusion that the global relative sea levels, possibly due to changes in atmospheric surface air temperature, could have had substantial impacts on the fluctuation of the Chinese white dolphin population.

### 2.3. Identification of AHTPs

**Figure 5:**
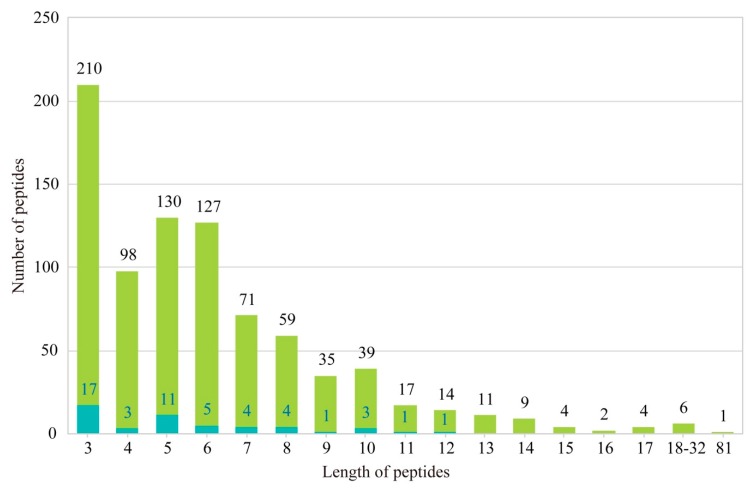
Statistic of antihypertensive peptides (AHTPs) in our local database and those employed for this study. Blue boxes within some groups denote the number of AHTP peptides explored in this study, which potentially have a higher activity for antihypertension based on previous reports.

In order to screen potential AHTPs in the deduced proteome of Chinese white dolphin, we established a local database (Table S4) for those AHTPs that have been verified in previous studies. In fact, most of them are tripeptides and usually less than 10 amino acids (Figure 5). 

We picked out the top 50 active AHTPs (Table S4) to map the deduced proteome of Chinese white dolphin and the downloaded protein datasets (Table S5), and identified 35 AHTPs in the five representative mammals (Table S9), including the terrestrial cow (34), marine minke whale (34), epipelagic Chinese white dolphin (31) and bottlenose dolphin (31), as well as the autopotamic Yangtze River dolphin (31). All the mapping results were listed in Table S6, and the hit numbers of AHTPs in each protein of the five examined mammals were sorted in Table S7. 

**Figure 6:**
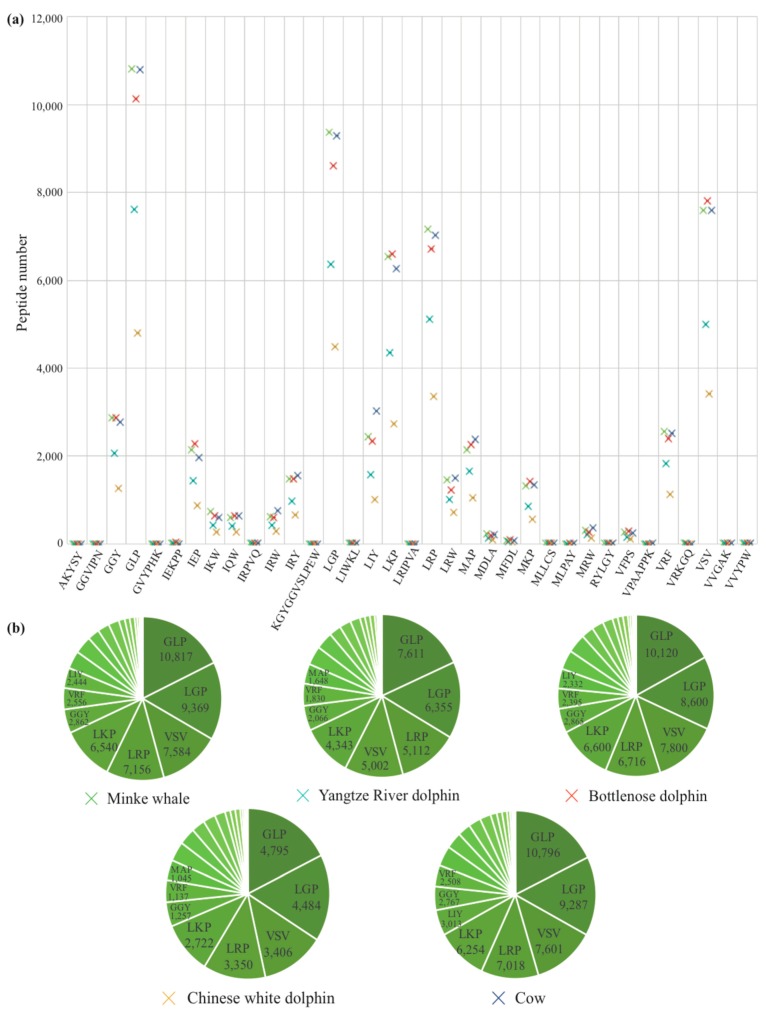
Comparison of each AHTP hit number in the five examined mammals (**a**) and summary of the top eight abundant AHTPs in each mammal species (**b**).

As shown in Table S9, the composition of AHTPs seems to determine their presence in the whole proteome datasets. In general, Leu-Gly-Pro (LGP) had a much higher frequency than Leu-Lys-Pro (LKP) and Leu-Arg-Pro (LRP) in the five examined mammals, while the occurrence of LRP was closer to LKP than to Leu-Arg-Trp (LRW). Among the 35 characterized AHTPs, Gly-Leu-Pro (GLP), LGP, Val-Ser-Val (VSV), LRP and LKP were the major components in the five mammal protein datasets (Table S9, Figure 6). There were fewer mapping results for longer peptides, including four peptides that only existed in the minke whale and/or terrestrial cow. Interestingly, for the most mapped AHTPs, the white dolphin had the least hit numbers (Table S9, Figure 6a), which is consistent with the least annotated gene/protein number among the five mammal genomes.

Minke whale and cow possessed the most abundant AHTP hits (60,820 and 61,028, respectively) and mapped proteins (25,079 and 25,012, respetively), while the Chinese white dolphin had the least with 27,260 hits in 18,387 mapped proteins (Table 2). However, their mapping rates were at a narrow range from about 0.62 to 0.67. Average AHTP numbers of all mapped proteins in the five mammals were also between 2.3 and 2.5. Our mapping results revealed that titin and collagen type IV alpha protein were at the top with the most abundant AHTP hits in these mammals (Table S7), which is consistent with our previous report in 18 fishes [34]. It therefore seems that the occurrence of AHTPs on their mapped protein sequences may be highly conserved among vertebrates, which may suggest conservation of AHTP-dependent antihypertensive mechanisms in various animals. 

Generally speaking, the minke whale mapped more collagen subunit proteins than the other four mammals, although the Chinese white dolphin had 48 mapped collagen subunits (Table 2). The longest peptide, KGYGGVSLPEW, isolated from whole whey proteins, was only identified in the lactalbumin alpha protein of cow in this study. Our data consolidate the reliability of our genomic approach in high-throughput discovery of bioactive peptides.

**Figure 7:**
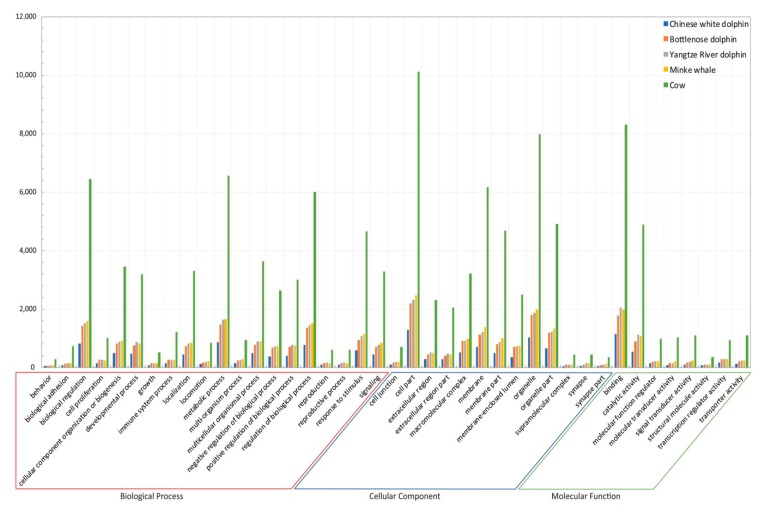
Functional annotation of AHTP-mapped proteins in the five examined mammals.

Functional annotation of AHTP-containing proteins revealed a similar distribution pattern in the five examined mammals (Figure 7, Table S8). However, the annotated protein number of cow in each functional category far more exceeded that of the others. It seems that their functions focused on metabolic process and regulation of biological process; that is, they were mainly components of cells, membranes and organelle parts, and possessed binding and catalytic activity in a molecular function term. These results proved that the categories of AHTP-containing proteins in the five mammals were much similar, which conforms to their similar mapping rates at the whole proteome level. Although these mammals are from different habitats, our comparisons of AHTPs and AHTP-containing proteins support the high conservation between aquatic and terrestrial animals.

## 3. Discussion

Previous researches have indicated that expanded gene families to some extent may reflect specific physiological adaptation and special traits in the studied lineages [35]. The cetaceans that live in offshore environments may often face many physiological challenges, such as sensory disruption. They have to evolve and reserve some special characters to adapt to various conditions. An expansion of gene families related to nervous and sensory systems was found in the Chinese white dolphin ([21] and this study), suggesting that this marine mammal has developed a very sensitive sensory system for anthropogenic or prey pressures. We also identified some expanded gene families involved in DNA replication and repairing, indicating that the Chinese white dolphin may have a great capacity to replicate and repair DNA due to the potential serious damages from a high level of UV stimuli in tropical regions and nearshore environments. These data are consistent with the previous genome report of the Chinese white dolphin [21]. 

Our genome assembly of the Chinese white dolphin also provides a valuable genetic resource for AHTP research in aquatic mammals for the first time. The comparisons in this study among the five representative mammals, including the terrestrial cow, marine minke whale, freshwater Yangtze River dolphin, as well as the epipelagic bottlenose dolphin and Chinese white dolphin, showed that AHTPs in these mammals were highly conserved. In our previous study of AHTPs in whole proteome datasets of 18 fish species [34], we also observed a conserved mapping pattern. 

The living conditions of these examined mammals have no obvious association with the amount of AHTPs. The mapping rate and average AHTP hit number of each mapped protein were similar in this study. Collagen subunit proteins among the longest proteins possess the top abundant AHTPs here (Table S7), especially for the type IV alpha 5 (col4a5) and type VIII alpha 1 (col8a1) subunits from both our studies of fish and mammals. Moreover, the most abundant AHTP categories involved GLP, LGP and VSV in the five examined mammals. We previously found that Atlantic salmon had the most abundant AHTPs in comparison with other fishes [34]. In this study, cow and minke whale had an equal high level of AHTP hit numbers. These AHTP-containing proteins were reported to participate in multiple biological and metabolic functions. Several mechanisms contribute to the maintenance of blood pressure, at steady state and during diving [36]. Thus, the richness of collagen subunit proteins in the minke whale (Table 2), the longest diving cetacean among the analyzed species, may suggest their importance towards adaptation to diving-induced hypoxia [37]. These proteins are also a potential resource for development of AHTP-based marine drugs. 

## 4. Materials and Methods

### 4.1. Sample Collection and Preparation

A cell line derived from the skin of a Chinese white dolphin was prepared as we reported previously [25]. The skin sample was collected by using a noninvasive method from a male adult individual, which was rescued for rehabilitation from an animal live-stranding event in a shallow river near Foshan City, Guangdong Province, China. Under the permission of the Pearl River Estuary Chinese White Dolphin National Nature Reserve (No. 2017A030308005), we obtained assistance from local veterinarians to collect samples. The sampling site on the dolphin’s back was sterilized by surgical cottons with 70% alcohol, and the tissue fragments were sheared off aseptically by scraping with a blade. The wound (approximately 0.2 cm^2^) was treated immediately with haemostatic and anti-inflammatory ointments. The skin tissue was immersed into Dulbecco’s modified Eagle’s medium (DMEM; ThermoFisher Scientific, Waltham, MA, USA) including penicillin (100 U/ml), streptomycin (100 μg/ml) and amphotericin B (5 μg/ml), and then immediately transported on ice within 2 h to our laboratory for a subsequent cell-line preparation [25]. 

Genomic DNA was extracted from cells using a Tissue DNA Kit (Omega, Norcross, GA, USA) according to the manufacturer’s protocol. All experiments were performed in accordance with the Regulations of the Animal Ethics Committee and were approved by the Institutional Review Board on Bioethics and Biosafety of Sun Yat-Sen University, China. 

### 4.2. Genome Sequencing and Assembling

We employed the traditional whole-genome shotgun sequencing strategy and constructed seven paired-end libraries with diverse insert sizes, including three short-insert libraries (270, 500 and 800 bp) and four long-insert libraries (2, 5, 10 and 20 kb), for genome sequencing of the extracted genomic DNA on an Illumina HiSeq 2500 platform. About 318.4 Gb of raw reads were generated. After removal of low-quality and redundant reads, we obtained 245.9 Gb of clean data for further de novo assembly (Table S1). 

We employed SOAP-denovo2 [38] (with -k 65) to build contigs and primary scaffolds by utilizing reads from the short-insert libraries (250, 500 and 800 bp). Subsequently, reads from the long-insert libraries (2, 5, 10 and 20 kb) were mapped onto contigs to shape corresponding scaffolds. Gapcloser in the package of SOAP-denovo2 was employed to fill the gaps within those achieved scaffolds. Our genome assembly of the Chinese white dolphin was deposited in NCBI with the accession number of RWJT00000000.

### 4.3. Genome Annotation

We first identified repeat sequences in our genome assembly using the Tandem Repeats Finder [39], LTR_FINDER [40], RepeatProteinMask and RepeatMasker (version 3.2.9, Institute for System Biology, Seattle, CA, USA) [41]. The Tandem Repeat Finder was employed to search the tandem repeats from the genome assembly with the following parameters: Match = 2, Mismatch = 7, Delta = 7, PM = 80, PI = 10, Minscore = 50 and MaxPerid = 2000. A de novo repeat library was built by the LTR_FINDER software (version 1.0.6, parameter: -w 2; University of Fudan, Shanghai, China). Subsequently, the RepeatMasker software was utilized to align our genome sequences onto the Repbase TE (version 3.2.9; Genetic Information Research Institute, Mountain View, CA, USA) [42] to search for known repeat sequences, which were also mapped onto the de novo repeat libraries to identify novel types of repeat sequences. 

We then annotated the *S. chinensis* genome assembly using three routine approaches, including homology-based, transcriptome-based and ab initio annotations. We selected several representative animal species to perform the homology annotation, including the Baiji dolphin (*Lipotes vexillifer*), sperm whale (*Physeter catadon*), bottlenose dolphin (*Tursiops truncates*), cattle (*Bos Taurus*), human (*Homo sapiens*), mouse (*Mus musculus*), sheep (*Ovis aries*), pig (*Sus scrofa*) and zebrafish (*Danio rerio*). Related protein sequences were aligned onto our genome sequences utilizing TblastN [43] with an E-value < 1.0e^−5^. Genewise 2.2.0 [44] was subsequently employed to predict possible gene structures based on the TblastN results.

Total RNA extracted from leucocytes was sequenced on an Illumina HiSeq 4000 platform. These transcriptome reads were aligned onto our genome assembly using HISAT [45]. We utilized Cufflinks (version 2.2.1; University of Maryland, College Park, MD, USA) [46] to identify the preliminary genes. Meanwhile, Augustus [47] and Genscan [48] were employed for ab initio annotation by using the repeat-masked genome sequences. Finally, we employed GLEAN [49] to integrate all predicted genes from the three annotation procedures. 

All protein sequences of the GLEAN results were mapped onto the public TrEMBL, SwissProt [50] and KEGG [51] databases using BLASTP with an E-value ≤ 1.0 e^−5^. We also applied the InterProScan [52] to predict potential functions of these protein sequences with Pfam [53], PRINTS [54], PANTHER [55], ProDom [56] and SMART [57]. 

### 4.4. Phylogenetic Relationships of the Chinese White Dolphin

To understand the phylogenetic relationships of Chinese white dolphin, we constructed a phylogenetic tree using Chinese white dolphin and other ten mammals, including six Cetacea species, i.e., Beluga whale (*Delphinapterus leucas*), Yangtze finless porpoise (*Neophocaena asiaeorientalis*), Baiji dolphin, bottlenose dolphin, sperm whale and minke whale (*Balaenoptera acutorostrata*)), as well as two Artiodactyla species (cattle and sheep) and two Euarchontoglires species (human and mouse). Whole-genome gene sets for the other ten mammals were available online, and thus we downloaded them from NCBI. These datasets and the gene sets predicted from the Chinese white dolphin genome were aligned with each other by BLAST (version 2.2.6; Genome Research Center, Cold Spring Harbor, NY, USA) [43] to determine homologous genes. In this way, we obtained 5728 single-copy gene families with 63,008 genes in total. These single-copy genes from each species were concatenated together to constitute a super-length gene that yielded 3,657,951 aligned sites. Finally, the four-fold degerated sites were extracted from these aligned sites to construct the phylogenetic tree with the maximum likelihood (ML) method using PhyML (version 3.0; Université de Montpellier, Montpellier, France) [58].

### 4.5. Molecular Dating

Bayesian molecular dating was adopted to estimate the neutral evolutionary rate and species divergence time with MCMCTREE from PAML (version 4.4b; University College London, London, UK) [59]. Five nodes (C1~C5) were considered as time-calibrated points with normal distributions and soft constraint bands, allowing a small probability (0.025) of violation. Based on previous research [35], we calibrated the phylogenetic tree with two time points, 61.7~100.5 Mya for Human–Mouse and 71.2–113 Mya for Laurasiatheria and Euarchontoglires, which were retrieved from the TimeTree database [60]. The divergence time between Cetacea and Artiodactyla using the Ypresian fossil Pakicetus (Eocene: 55.8–48.6 Mya) [61,62] was applied as the C3 calibrate point; the C4 calibration point was 10~30 Mya for sheep and cattle based on a previous report [27]; and the divergence between Phocoenidae (such as Finless porpoise) and Monodontidae (such as Beluga whale), calibrated on the basis of the earliest record of a Phocoenid (10.0~11.2 Mya) [63], was chosen as the C5 calibration point. The MCMC (Markov chain Monte Carlo) chain length was set to 200,000 generations and sampled every 200. The first 20% of samples were discarded as burns in. An independent rate model (clock = 2) following a lognormal distribution was employed for the MCMC search.

### 4.6. Identification of Gene Family Changes

To determine the evolutionary dynamics of gene families, especially for expansion and contraction of gene ortholog clusters, we employed the program CAFÉ [64] to identify gene family changes among the Chinese white dolphin, Beluga whale, finless porpoise, Baiji dolphin, bottlenose dolphin, sperm whale, minke whale, cattle, sheep, human and mouse. 

### 4.7. Prediction of Historical Population

We realigned the reads from short-insert libraries (500 and 800 bp) onto our genome assembly using the package SOAP [65]. Subsequently, we called heterozygous SNPs (single nucleotide polymorphisms) using the package SOAPsnp [66] with an optimized threshold (coverage depth ≥ 4 and ≤ 150, genotype quality ≥ 20, copy number ≤ 2 and distance of adjacent SNPs ≥ 5). We then employed the Hidden Markov model (HMM) approach to implement Pairwise Sequentially Markovian Coalescence (PSMC) on the basis of SNP distribution [31]. 

We used these heterozygous SNPs to reconstruct a demographic history. The generation time (g = 12 years) and neutral mutation rate per generation (μ = 1.5 × 10^−8^) were based on a previous report [35]. We obtained atmospheric surface air temperature (°C) and global relative sea level (10 m) data of the past one million years from National Climatic Data Center (NCDC), and then combined them together with the demographic data to generate a single plot. However, it is difficult for the PSMC simulation to detect population changes within 10,000 years ago. We hence didn’t predict historical population during this recent period.

### 4.8. Identification of AHTPs

The top 50 previously reported AHTPs with high antihypertensive activities (Table S2) were selected to map our target protein datasets. These protein datasets were downloaded from NCBI and Ensemble for four mammals with different living habitats (Table S3), including cow (on land), minke whale (in deep sea), Yangtze River dolphin (in freshwater), and Bottlenose dolphin (in shallow sea). In-house scripts were compiled to identify AHTP-mapped proteins by in situ mapping, and the localization of each target peptide was marked for further statistics and analysis.

## 5. Conclusions

In summary, we report a high-quality genome with a relatively complete gene set for the Chinese white dolphin, an endangered nearshore marine mammal in China. Expansion of certain gene families, especially the increase in immune and sensory genes, could partly shed light on the molecular mechanisms for adaptation to the nearshore environments. We also observed a serious bottleneck in the demographic population history of Chinese white dolphin about 350,000 years ago. The identification of AHTPs broadens our knowledge about the potential of mammal proteins for development of antihypertensive peptides. Our genome assembly will provide a genetic resource for further researches on adaptive ecology, conservation biology of cetaceans and development of marine peptide-based drugs for treatment of various human cardiovascular diseases.

## Figures and Tables

**Table 1:** Summary of the assembly and annotation of the Chinese white dolphin genome.

**Genome assembly**	**Parameter**
Contig N50 (kb)	84.3
Scaffold N50 (Mb)	19.2
Assembled genome (Gb)	2.3
Genome coverage (×)	318.4
Longest scaffold (bp)	71,519,079
**Genome annotation**	**Parameter**
Number of protein-coding genes	18,387
Transposable elements content (%)	42.3

**Table 2:** Summary of the AHTP mapping results in the five representative mammals.

Parameter	Minke Whale	Yangtze River Dolphin	Bottlenose Dolphin	Chinese White Dolphin	Cow
Total hits	60,820	41,733	58,992	27,260	61,028
Mapped protein	25,079	17,633	25,593	11,323	25,012
Total protein	37,625	26,901	38,849	18,387	37,525
Annotated protein number	3206	3105	2768	1692	13,435
Mapping rate	0.6666	0.6555	0.6588	0.6158	0.6665
Average AHTPs number in mapped protein	2.4251	2.3668	2.3050	2.4075	2.4399
Collagen subunit number in mapped protein	92	66	69	48	75

## Supplementary Materials

The following materials are available online at https://www.mdpi.com/1660-3397/17/9/504/s1. Figure S1: Molecular dating of the Chinese white dolphin and other ten examined mammals. Table S1: Statistics of raw reads and clean data for the whole genome sequencing. Table S2: Summary of repeat sequences in the assembled genome. Table S3: Statistics of functional annotations. Table S4: A local database of AHTPs. Table S5: Summary of the downloaded protein datasets for other four mammals. Table S6: Mapped AHTPs in the five mammalian proteome datasets. Table S7: Hit numbers of AHTPs in the five examined mammals. Table S8: GO annotation of AHTP-mapped proteins in the five examined mammals. Table S9: Numbers of each mapped AHTP in the five examined mammals.
