# Supplementary material for: Whole Genome Sequencing of Chinese White Dolphin (Sousa chinensis) for High-Throughput Screening of Antihypertensive Peptides
Source: Mar Drugs. 2019 Aug 28;17(9):504. doi: 10.3390/md17090504 (PMC6780146; doi:10.3390/md17090504)
Supplement: Supplementary file 1 [file marinedrugs-17-00504-s001.zip › Supplementary Fig1 & Table S1-3,5,9 revised.docx]

**Supplementary Figures**

**
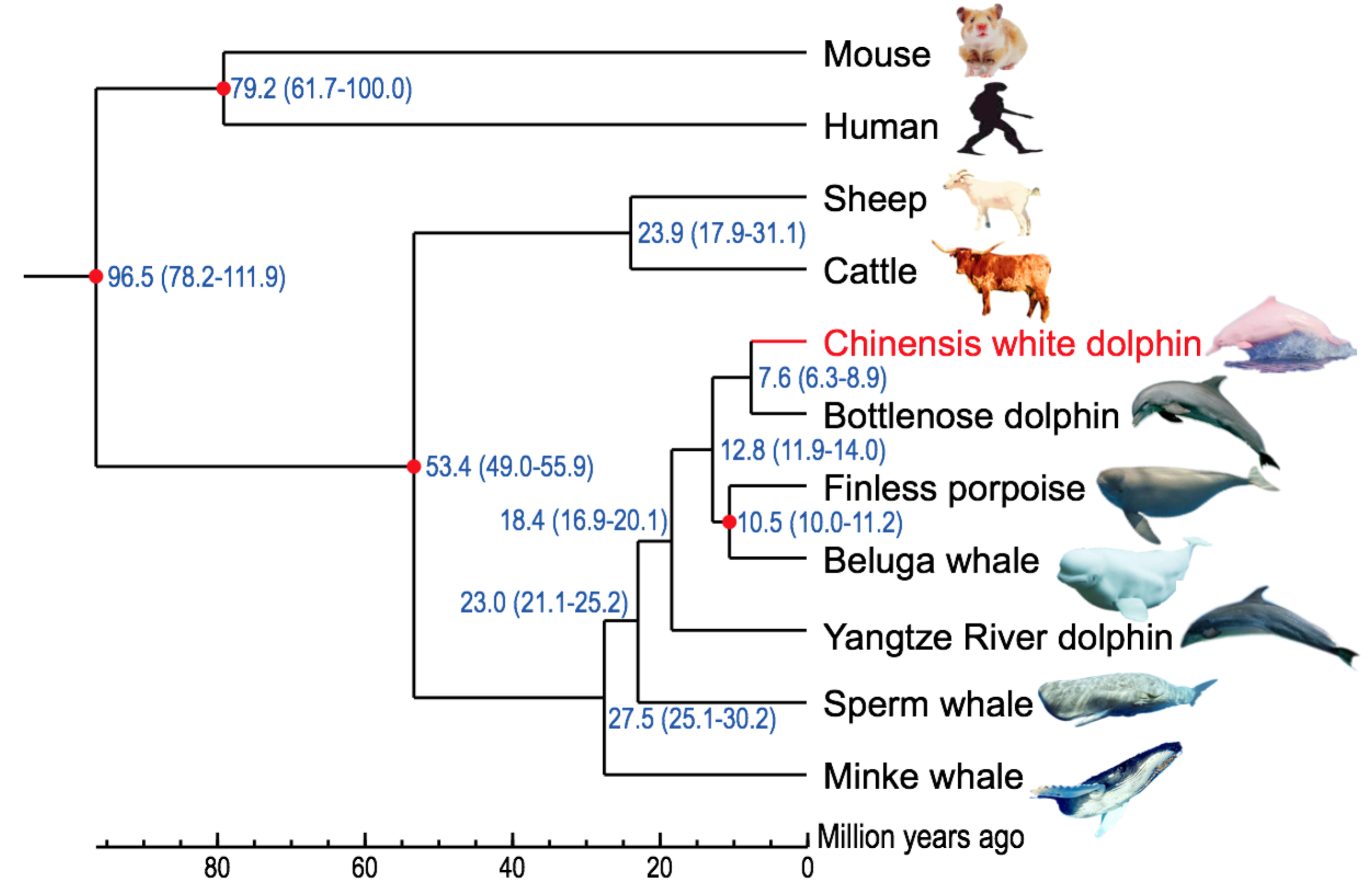
**

**Figure S1.** Molecular dating of the Chinese white dolphin and other ten examined mammals. The red points of C1~C5 in the branches stand for five calibrated time points (with two fossil points), which were used in our present study.

## Supplementary Tables

**Table S1.** Statistics of raw reads and clean data for the whole genome sequencing.

| **Data type** | **Insert Size**  **(bp)** | **Read Length**  **(bp)** | **Data Size**  **(Gb)** |
| --- | --- | --- | --- |
| Raw data | 270 | 150 | 119.2 |
|  | 500 | 125 | 31.1 |
|  | 800 | 125 | 16.9 |
|  | 2,000 | 125 | 33.2 |
|  | 5,000 | 125 | 46.3 |
|  | 10,000 | 125 | 35.5 |
|  | 20,000 | 125 | 36.3 |
| Total |  |  | 318.4 |
| Clean data | 270 | 145 | 99.7 |
|  | 500 | 120 | 28.9 |
|  | 800 | 120 | 16.0 |
|  | 2,000 | 120 | 25.5 |
|  | 5,000 | 120 | 33.1 |
|  | 10,000 | 120 | 23.0 |
|  | 20,000 | 120 | 19.7 |
| Total |  |  | 245.9 |

**Table S2.** Summary of repeat sequences in the assembled genome.

| **Type** | **Method** | **Repeat Size (bp)** | **% of Genome** |
| --- | --- | --- | --- |
| Tandem Repeats | TRF | 51,501,384 | 2.17 |
| Transposable elements  (TEs) | Repeatmasker | 852,477,295 | 36.04 |
|  | Proteinmask | 231,894,303 | 9.80 |
|  | *De novo* | 800,117,782 | 33.83 |
| Total |  | 999,680,059 | 42.27 |

**Table S3.** Statistics of functional annotations.

| **Parameter** | **Total** | **Swissprot** | **KEGG** | **TrEMBL** | **Interpro** | **Overall** |
| --- | --- | --- | --- | --- | --- | --- |
| Number | 18,387 | 17,003 | 14,993 | 17,228 | 16,867 | 17,268 |
| Percentage | 100% | 92.47% | 81.54% | 93.70% | 91.73% | 93.91% |

**Table S4.** A local database of AHTPs (see a separate excel file).

**Table S5.** Summary of the downloaded protein datasets for other four mammals.

| **Species** | **Common name** | **Habitats** | **Sequence Number** | **Websites** |
| --- | --- | --- | --- | --- |
| *Bos taurus* | Cow | Land | 37,525 | ftp://ftp.ensembl.org/pub/release-95/fasta/bos_taurus/pep/ |
| *Balaenoptera acutorostrata* | Minke whale | Deep sea | 37,625 | ftp://ftp.ncbi.nlm.nih.gov/genomes/all/GCF/000/493/695/GCF_000493695.1_BalAcu1.0/ |
| *Lipotes vexillifer* | Yangtze River dolphin | Shallow freshwater | 26,901 | ftp://ftp.ncbi.nlm.nih.gov/genomes/all/GCF/000/442/215/GCF_000442215.1_Lipotes_vexillifer_v1/ |
| *Tursiops truncatus* | Bottlenose dolphin | Shallow sea | 38,849 | ftp://ftp.ncbi.nlm.nih.gov/genomes/all/GCF/001/922/835/GCF_001922835.1_NIST_Tur_tru_v1/ |

**Table S6.** Mapped AHTPs in the five mammalian proteome datasets (see a separate excel file).

Table S7: Hit numbers of AHTPs in the five examined mammals (see a separate excel file).

Table S8: GO annotation of AHTP-mapped proteins in the five examined mammals (see a separate excel file).

Table S9: Numbers of each mapped AHTP in the five examined mammals.

| **Peptide** | **Minke whale** | **Yangtze River dolphin** | **Bottlenose dolphin** | **Chinese white dolphin** | **Cow** |
| --- | --- | --- | --- | --- | --- |
| AKYSY | 1 | 4 | 4 | 2 | 3 |
| GGVIPN | 1 | 0 | 0 | 0 | 0 |
| GGY | 2,862 | 2,066 | 2,865 | 1,257 | 2,767 |
| GLP | 10,817 | 7,611 | 10,120 | 4,795 | 10,796 |
| GVYPHK | 1 | 0 | 0 | 0 | 1 |
| IEKPP | 25 | 11 | 26 | 7 | 5 |
| IEP | 2,147 | 1,448 | 2,290 | 884 | 1,963 |
| IKW | 736 | 429 | 648 | 270 | 606 |
| IQW | 611 | 411 | 639 | 265 | 645 |
| IRPVQ | 10 | 8 | 14 | 4 | 9 |
| IRW | 624 | 432 | 603 | 283 | 765 |
| IRY | 1,471 | 982 | 1,487 | 662 | 1,566 |
| KGYGGVSLPEW | 0 | 0 | 0 | 0 | 4 |
| LGP | 9,369 | 6,355 | 8,600 | 4,484 | 9,287 |
| LIWKL | 11 | 11 | 8 | 2 | 6 |
| LIY | 2,444 | 1,573 | 2,332 | 1,011 | 3,013 |
| LKP | 6,540 | 4,343 | 6,600 | 2,722 | 6,254 |
| LRIPVA | 3 | 2 | 3 | 2 | 2 |
| LRP | 7,156 | 5,112 | 6,716 | 3,350 | 7,018 |
| LRW | 1,461 | 1,018 | 1,220 | 721 | 1,504 |
| MAP | 2,137 | 1,648 | 2,263 | 1,045 | 2,377 |
| MDLA | 225 | 133 | 198 | 98 | 207 |
| MFDL | 67 | 58 | 98 | 31 | 77 |
| MKP | 1,315 | 847 | 1,427 | 565 | 1,348 |
| MLLCS | 16 | 9 | 16 | 7 | 22 |
| MLPAY | 3 | 5 | 15 | 4 | 14 |
| MRW | 311 | 203 | 274 | 124 | 361 |
| RYLGY | 16 | 9 | 11 | 4 | 13 |
| VFPS | 267 | 149 | 291 | 106 | 244 |
| VPAAPPK | 1 | 0 | 0 | 0 | 9 |
| VRF | 2,556 | 1,830 | 2,395 | 1,137 | 2,508 |
| VRKGQ | 13 | 8 | 3 | 4 | 3 |
| VSV | 7,584 | 5,002 | 7,800 | 3,406 | 7,601 |
| VVGAK | 11 | 10 | 20 | 5 | 19 |
| VVYPW | 8 | 6 | 6 | 3 | 11 |
